# Ten tips for a text-mining-ready article: How to improve automated discoverability and interpretability

**DOI:** 10.1371/journal.pbio.3000716

**Published:** 2020-06-01

**Authors:** Robert Leaman, Chih-Hsuan Wei, Alexis Allot, Zhiyong Lu

**Affiliations:** National Center for Biotechnology Information (NCBI), National Library of Medicine (NLM), National Institutes of Health (NIH), Bethesda, Maryland, United States of America

## Abstract

Data-driven research in biomedical science requires structured, computable data. Increasingly, these data are created with support from automated text mining. Text-mining tools have rapidly matured: although not perfect, they now frequently provide outstanding results. We describe 10 straightforward writing tips—and a web tool, PubReCheck—guiding authors to help address the most common cases that remain difficult for text-mining tools. We anticipate these guides will help authors’ work be found more readily and used more widely, ultimately increasing the impact of their work and the overall benefit to both authors and readers. PubReCheck is available at http://www.ncbi.nlm.nih.gov/research/pubrecheck.

## Abstract 2

Your published research is already being processed with automated tools, and text mining will become more common; this Community Page article describes how you can help these tools process your work more accurately, including a web tool, PubReCheck.

## Introduction

Automated text analysis has proven very effective for helping researchers search the biomedical literature to retrieve relevant articles [1,2]. But as biomedical research becomes increasingly quantitative, the requirement for data-driven research is pushing a need for more specific knowledge embedded within individual articles and for more comprehensive results across the literature [3]. Biocuration addresses these needs by manually extracting the unstructured information in free text articles into structured and computable data in knowledge bases [4]. These curated resources enable connections between seemingly disparate studies and have become essential to current biomedical research. However, data curation at scale remains challenging because of the requirement for significant manual effort by humans [5,6]. Text mining can greatly complement human efforts by automating the conversion of unstructured text such as scientific publications into structured, computable formats, thereby enabling more rapid analyses at a larger scale [7]. Successful uses of text mining in biology include literature-based knowledge discovery [8–11], facilitating analysis of high-throughput (gene expression/genome-wide association) data [12,13], detecting false and contradictory findings [14], and pharmacovigilance [15], among many others.

**Fig 1:**
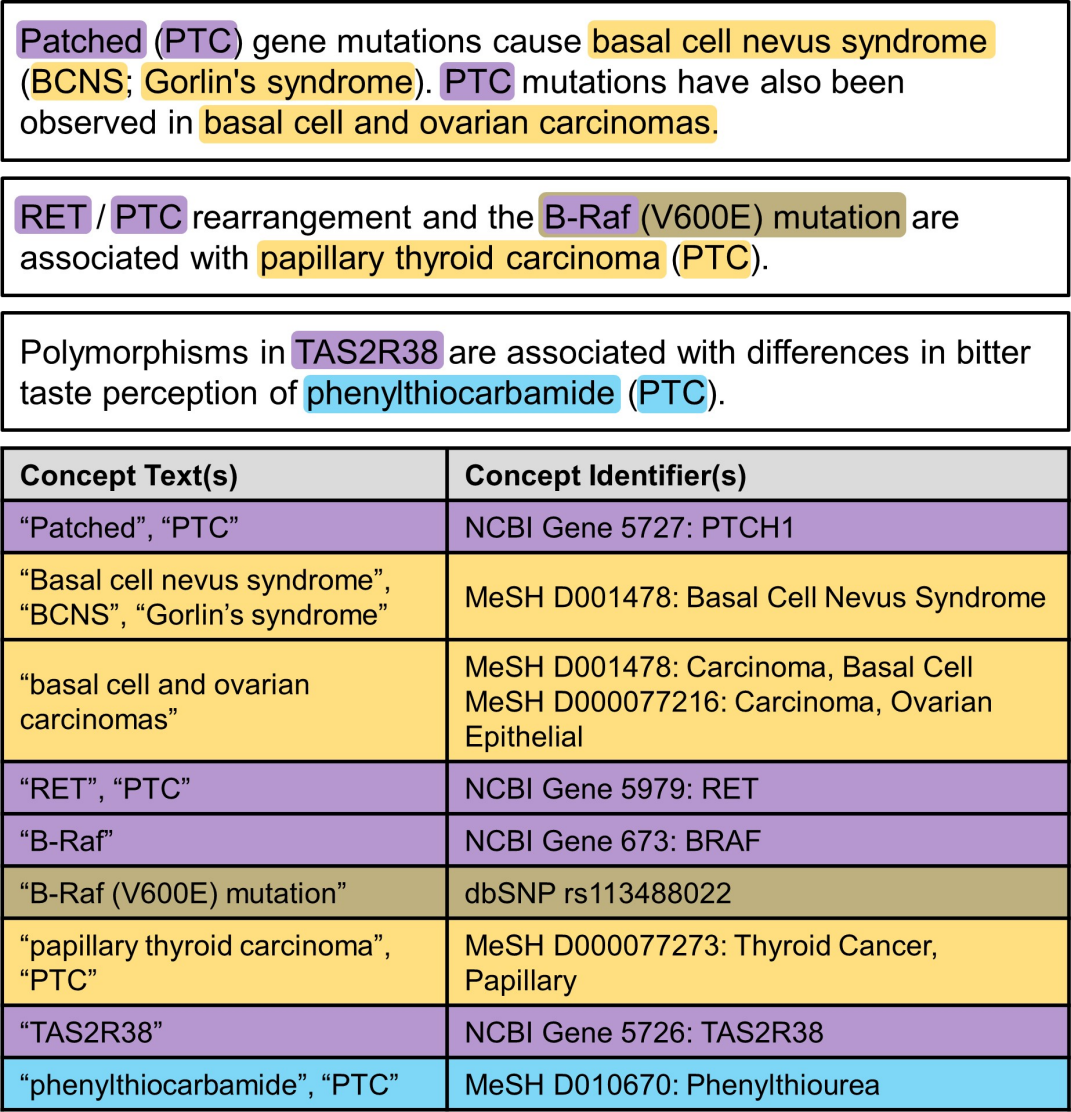
Concept extraction is a critical step in text mining. Phrases referring to a concept are associated with an identifier from an appropriate database (purple = gene, orange = disease, brown = mutation = brown, cyan = chemical). Text-mining systems must handle variation—“patched” and “PTC” both refer to “PTCH1”—and ambiguity—“PTC” could refer to “PTCH1,” “RET,” “papillary thyroid carcinoma,” or “phenylthiourea.” Examples adapted from [20–22].

A critical step in almost all text-mining systems is identifying words and phrases within the text that refer to biomedical concepts. This long-standing task in biomedical text mining was first considered in the 1990s [16] and has been addressed at a number of community-wide challenges since 2004 [17]. The extracted text is then linked with concepts from the relevant biological databases or controlled vocabularies, making the content more accessible, especially to large-scale computational analysis. Fig 1 illustrates concept extraction using extracts from three PubMed articles. Concept recognition systems have matured significantly, identifying a variety of biomedical concepts [18] with performance approaching that of an individual human annotator [19]. Despite significant progress, however, the accuracy of text-mining results remains imperfect.

For text mining to realize its full potential as a powerful method for “seeking a new biology” [23], it is imperative that the critical step of concept extraction be performed as accurately as possible. As illustrated in Fig 1, concept extraction is difficult because of variation and ambiguity, both of which are present to some degree in any natural language. Variation allows concepts to be referenced in multiple ways; for example, Fig 1 shows that the human gene “PTCH1” can also be called “patched” or “PTC,” the condition “basal call nevus syndrome” is also known as “Gorlin’s syndrome,” and “phenylthiourea” and “phenylthiocarbamide” are synonyms. Ambiguity, on the other hand, allows a single phrase to refer to multiple concepts; for example, the acronym “PTC” might refer either to the PTCH1 or RET genes, to papillary thyroid carcinoma, or to phenylthiourea. Managing variation and ambiguity is an important goal of terminology standardization efforts [24,25]. Although we strongly support standardization, we also recognize that authors may not find it practical to identify and apply all relevant standards.

The imperfection of existing automated methods and the difficulty of standardizing terminology has motivated active discussion of alternatives. One proposal suggests that the authors themselves should identify the biomedical concepts referenced in their article [26]. Under this proposal, authors would use controlled vocabularies or ontologies to identify the concepts mentioned in their article prior to publication, similar to the requirement to submit new genes to a central database prior to publication of the manuscript [23]. Unfortunately, this approach requires authors to become knowledgeable in terminologies and curation, which may not be practical. Another proposal is crowdsourcing, in which tasks are outsourced and distributed to many nonexpert workers online, typically by decomposing large tasks into individual decisions [27]. Despite progress, neither alternative has succeeded to date at a large scale.

Meanwhile, recent advances in natural language processing and machine learning continue to improve automated text-mining systems, allowing them to reach an overall accuracy of approximately 80% or higher in many cases. Performance at this level provides mostly outstanding results, with additional help typically only required for the few cases that remain difficult. In this work, we propose 10 writing tips based on a comprehensive analysis of the most prevalent errors experienced by current approaches for automatic concept extraction. We have attempted to make these tips straightforward for authors to implement—regardless of their preferred English dialect—and believe that the suggestions should typically also make the text clearer to human readers. We also provide a web-based tool, PubReCheck (http://www.ncbi.nlm.nih.gov/research/pubrecheck), to help authors visualize the information automated concept extraction tools derive from their text and to automatically identify many types of issues prior to publication. Published research that follows these guides will typically be processed more accurately by automated text analysis tools. We anticipate these guides will allow the author’s work to be found more readily and used more widely, ultimately helping the millions of readers who search the biomedical literature satisfy their information needs.

## Top 10 Tips

### Tip 1: Clearly associate gene and protein names with the species

Entries in gene and protein databases are differentiated by species: in humans, the BRCA1 gene is NCBI Gene 672, but in mice (*Mus musculus*) it is NCBI Gene 12189. Automated systems for identifying genes and proteins must, therefore, determine the species first. If the species is not mentioned directly, the system must try to infer it from related concepts: the cell line “GH(3)” implies *Rattus norvegicus* [28] and the strain “TA100” signifies *Salmonella typhimurium* [29]. Although inferring the species from context is often effective, directly stating the species significantly reduces the potential for error. We recommend authors clearly mention the relevant species when discussing genes or proteins whenever possible. This is especially important the first time it is mentioned: for example, “we investigated the role of Brca1 during mouse embryonic cortical development …” [30]. This recommendation does not apply, however, in cases when the discussion is not with respect to any specific species, such as when referring to (homologous) genes in a general context.

### Tip 2: Supply context critical for comprehension prominently and in proximity

Like human readers, text-mining systems use the surrounding context to help resolve ambiguous words and phrases. For example, whereas “p24” could refer to at least four different human proteins, the text “p24 also maps to chromosome 12” [31] must refer to the product of human gene TMED2 (NCBI Gene 10959), because the other possibilities are assigned to other chromosomes. Synonyms are especially useful for clarifying the intended meaning; for example, the synonym provided in the text, “which interacts with human NAP1 (NCKAP1) …” [32], narrows the number of possibilities for “NAP1” from 9 to 1 (NCBI Gene 10787). The need for clarifying context may arise because a specific name is ambiguous, or it may arise because a specific relationship is required, such as the species for a gene or a gene name for a genetic variant.

Although context can resolve ambiguity effectively, it is only helpful if the correct association can be identified. This is especially important for the abstract: it summarizes the article, is often provided without the full article text, and is the focus of many text-mining methods—though work on mining full-text articles is well underway [33–35]. Current automated methods for context association are more accurate when the related information is within the same sentence or, at most, within the same paragraph. Although the abstract cannot contain the details necessary to analyze its claims, if comprehending the summary provided by the abstract requires context from other sections—for example, to determine which “p24” protein is the subject of the article—then the risk of error is significantly increased. We recommend authors provide context that is critical for comprehension (such as identifying ambiguous concept names) prominently—in the abstract—and in proximity, preferably in the same sentence.

### Tip 3: Define abbreviations and acronyms

Abbreviations and acronyms allow cumbersome terms to be referenced more concisely: “acute myeloid leukemia” could become “AML.” Although a few carefully chosen acronyms can improve readability, acronyms can also be ambiguous because they typically have more than one possible meaning. For example, “AML” often refers to “acute myeloid leukemia,” but it is also used for “angiomyolipoma,” “anterior mitral leaflet,” “amlodipine,” “amoxicillin,” and “amiloride.” The intended meaning of an acronym is therefore usually provided at first use, as in “Patients with acute myeloid leukemia (AML) are …” [36]. Without such a definition, a human reader or text-mining system must infer the meaning of an acronym from context, which is often unclear. We recommend all abbreviations and acronyms be listed with the corresponding full term the first time they are used.

### Tip 4: Refer to concepts by name

Language is variable: it can communicate ideas in multiple ways. Accordingly, a text might refer to a concept by name (“orthostatic hypotension”) or with a description (“immediate drop in systolic blood pressure observed on standing” [37]). Descriptions can be very helpful, but names provide several important advantages for automated tools. Names are usually easier for automated tools to locate because they have a simpler structure and less freedom for variation than descriptions. This makes them easier to differentiate from the surrounding text and match against the names in a controlled vocabulary, thus identifying them as referring to the corresponding concept. Names are also shorter than descriptions and thus have fewer places where the reference could potentially begin or end, which translates to fewer opportunities for error. We recommend referring to concepts primarily by name; when a description is needed, we suggest providing both.

### Tip 5: Use one term per concept

Synonyms can help authors clarify their intended meaning when a concept is first introduced, as in “The mouse Foxq1 gene, also known as Hfh1 …” [38] or “Primary hyperaldosteronism (Conn's syndrome) is …” [39]. However, using multiple terms interchangeably without clearly indicating that they should be considered equivalent is confusing at best and at worst may cause the reader—or text-mining system—to consider them to refer to different concepts. For automated algorithms, even minor variations such as hyphenation (“gastroenteritis” vs “gastro-enteritis” [40]) or possessives (“Schiff bases” vs “Schiff’s bases” [41]) require additional handling, increasing the risk of error. We recommend authors choose a term for each concept and use it consistently—varying from the exact text chosen only when required, such as for capitalization or plurals.

### Tip 6: Prefer the complete and precise term

Some biomedical terms have multiple meanings and are therefore ambiguous, despite being commonly used in the literature. The term “yeast” often refers specifically to the model organism *Saccharomyces cerevisiae*, but there are over 1,500 known species of yeast—one of which (*Candida auris*) is an emerging global health threat [42]. Similarly, “mouse” frequently refers to *M*. *musculus*, but “mouse” could refer to any species in the genus or to the genus itself. Subtypes also matter: despite some shared characteristics, “type 1 diabetes” and “type 2 diabetes” have many clinically relevant differences. Other ambiguous terms involve a close relationship between concepts. For example, “Epstein-Barr” probably refers to either to “Epstein-Barr virus” or “Epstein-Barr infection” and “Multiple Endocrine Neoplasia Type 1 (MEN1)” refers to “MEN1 syndrome” or “MEN1 gene.” We recommend preferring the precise and complete scientific term wherever possible. If the full term is too cumbersome, we suggest clarifying a more convenient term at first use, as in “The laboratory rat (Rattus norvegicus) is …” [43]. However, detailed classifications that are irrelevant or uncertain should be withheld, as in “Danio sp. could therefore play a significant role in controlling mosquito breeding …” [44].

### Tip 7: Coordinate compound terms cautiously

Compound terms such as “pineal tumour” and “retinal tumour” are often combined or coordinated to form a single phrase, as in “pineal and retinal tumours” [45]. Although simple coordinated phrases often improve readability, the number of possible interpretations for complex coordinated phrases quickly make them difficult to interpret. For example, a human reader might be able to determine that the phrase “unstimulated and MTb- or LPS-stimulated THP-1 cells” [46] refers to “unstimulated THP-1 cells,” “MTb-stimulated THP-1 cells,” and “LPS-stimulated THP-1 cells.” However, an automated tool must consider a vastly greater number of possibilities—such as “unstimulated LPS-stimulated THP-1 cells” and “MTb-1 cells”—and discard them as nonsensical. The risk that an automated tool will incorrectly prefer the wrong interpretation therefore depends strongly on the complexity of the coordinated phrase [47,48]. Simplifying the phrase—for example, to “unstimulated, MTb-stimulated, and LPS-stimulated THP-1 cells”—greatly reduces the complexity and thus the opportunities for error. We recommend that authors avoid creating coordinated compound terms with multiple potential interpretations.

### Tip 8: Spacing matters

Automated text-mining algorithms often initially identify meaningful units of text (such as sentences and words) and then proceed to interpret each unit. This approach is efficient and generally effective but tends to encounter problems if boundary markers such as spaces or periods are misplaced or missing. For example, the space missing from “ipratropiumbromide” [49] will make it more difficult to automatically recognize that it refers to the drug “ipratropium bromide.” Similarly, the space missing in “BRAFV600E” [50] will increase the difficulty of identifying the gene "BRAF" and the mutation "V600E." Extraneous spaces (“malignant lym phoma,” [51]) cause similar issues. We recommend identifying spacing issues with careful proofreading and spell-checking.

### Tip 9: Verify parentheses and brackets are correctly paired

Missing parentheses are also a concern, as in “ultrafiltration during cardiopulmonary bypass ICPB) has mandated …” [52]. Because parenthetical text is typically used to provide readers with information outside of the main narrative [53], correctly interpreting text with mismatching parentheses is difficult. Moreover, it is not straightforward for automated systems to determine whether a parenthesis should be added (and if so, which location) or removed (and if so, which one). We recommend verifying that all parentheses are correctly paired and no parentheses are placed extraneously.

### Tip 10: Recheck spelling with a different method

Biomedical terminology can be difficult to spell correctly, and it is not hard to find misspellings in the published literature. For example, not only does PubMed contain many examples of “hemorrhage” misspelled as “hemmorhage,” it also contains the plural (“hemmorhages”) and at least two related forms (“autohemmorhage” and “microhemmorhage”). Although automated tools can help with misspellings, the need to balance correcting errors against the possibility of introducing new ones makes automatic spelling correction more difficult than suggesting potential corrections to the user. On the other hand, recent text-mining tools should be able to handle true spelling variations (e.g., “leukemia” versus “leukaemia”).

Although careful proofreading is always valuable, proofreading manually may be subject to diminishing returns. We recommend rechecking for misspellings using a method not used previously. For example, authors could request assistance from a colleague not previously involved or use a different spell-checking tool. If nothing else, we suggest carefully rechecking each word in the title and abstract for any remaining spelling errors.

## Automated Tool: PubReCheck

**Fig 2:**
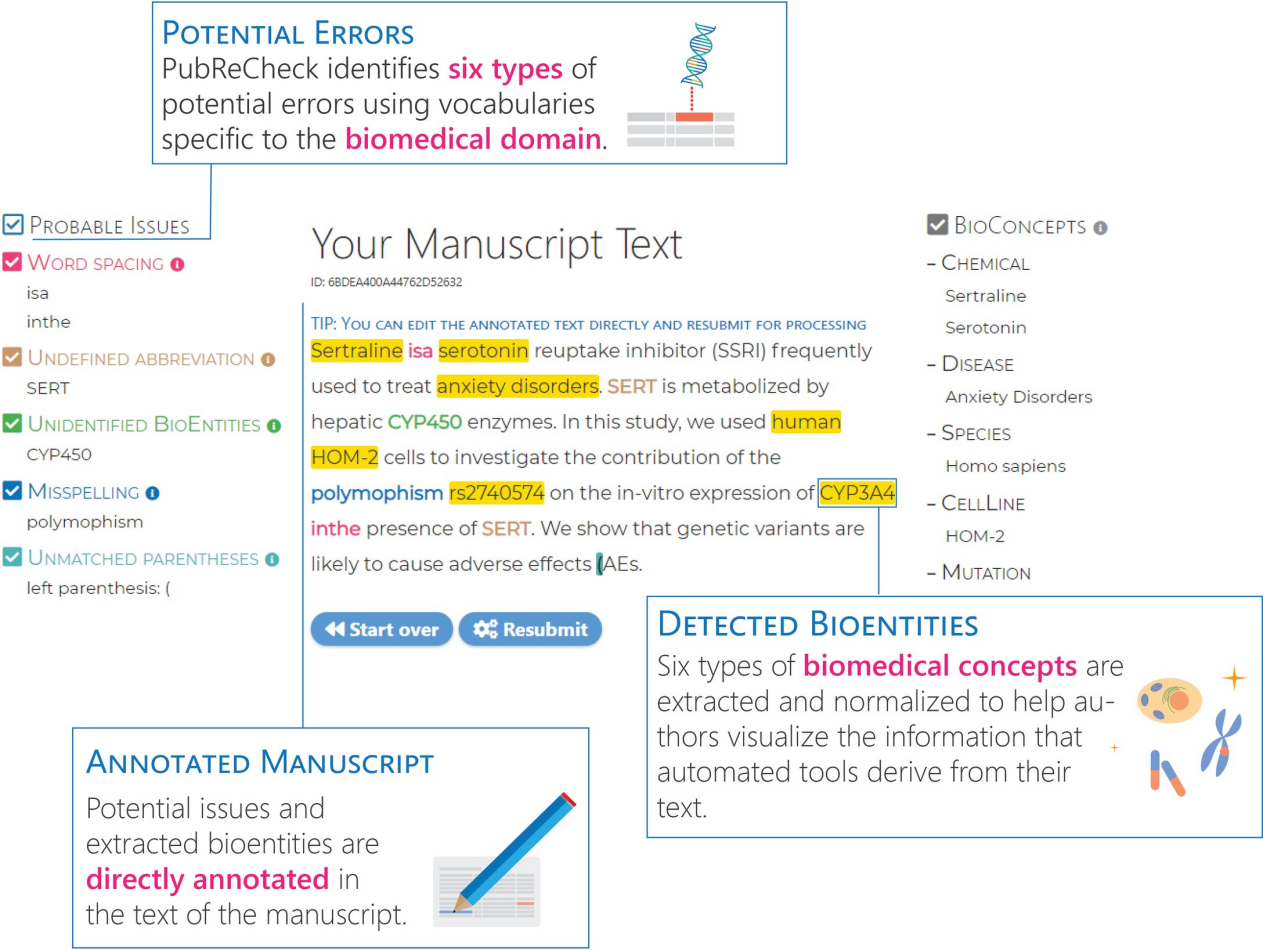
Screenshot of the PubReCheck system using an artificial abstract. PubReCheck identifies six types of biomedical concepts: genes, diseases, chemicals, genetic variants, species, and cell lines. PubReCheck also identifies six types of potential issues: misspellings, word spacing errors, undefined abbreviations, entities that cannot be uniquely identified, novel words, and unmatched parentheses.

To help authors automatically identify many types of issues prior to publication, we developed a web-based tool, PubReCheck (http://www.ncbi.nlm.nih.gov/research/pubrecheck). PubReCheck provides two primary functions, as shown in the screenshot of its results on a synthetic abstract in Fig 2. First, PubReCheck identifies six types of biomedical concepts: genes, diseases, chemicals, genetic variants, species, and cell lines. These results help authors visualize the information that automated tools derive from their text, allowing the text to be rephrased if required. PubReCheck also directly identifies six types of potential errors: misspellings, word spacing errors, novel words, undefined abbreviations, entities that cannot be uniquely identified, and unmatched parentheses. Like many automated tools, PubReCheck identifies misspellings (Tip 10) but is adapted to handle biomedical vocabulary that is rare in the general domain. Similarly, PubReCheck identifies potential word spacing errors (Tip 8)—phrases that need a space added or removed—and words that are uncommon in the biomedical literature, even if they may not be marked as a misspelling. PubReCheck also identifies undefined abbreviations (Tip 3) and unmatched parentheses and brackets (Tip 9). Finally, PubReCheck locates biomedical concepts that cannot be uniquely identified, which addresses several issues that may result in an ambiguous concept name (Tips 1, 2, and 6, primarily).

Automatically identifying and correcting potential errors is itself subject to errors. Error-checking the existing biomedical literature directly is problematic because it is difficult to recover from the additional errors that may be introduced. PubReCheck instead provides feedback directly to authors, who have both a strong interest in ensuring no errors remain and the ability to simply ignore a few phrases incorrectly identified as containing an error. PubReCheck therefore intentionally prioritizes identifying more potential errors. Moreover, because PubReCheck also allows authors to correct issues prior to publication, the maximum benefit is provided to both authors and readers.

## Conclusion

The continued rapid expansion of the biomedical literature necessitates the use of automated methods to address the information overload. Moreover, the increase in quantitative research in biology motivates moving beyond retrieving articles to extracting and converting their content to structured formats that enable computational processing. Although the accuracy of text-mining methods has improved dramatically in recent years—and will likely continue to improve—several issues remain difficult to address automatically.

Complementary to calls and initiatives that ask authors to follow standards and use standardized terminology, we have proposed a set of straightforward writing tips, summarized in Box 1, to help authors provide the information necessary to help automated text-mining algorithms to process their articles correctly. These tips—and especially our online tool, PubReCheck—may also be useful for editors, reviewers, publishers, and proofreaders. Although additional suggestions (and exemptions) could be identified, the ones presented have been chosen as likely to provide significant benefit for relatively modest effort. Articles that follow these tips will typically be processed more accurately, allowing their content to be found more readily and used more widely, thereby increasing its impact. Following these guidelines at a large scale will improve the ability of individual researchers to find the articles that meet their information needs. In short, following these tips will help us help you, and millions.

**Box 1. Summary of our recommendations to help articles be processed more accurately**

- Clearly mention the relevant species when discussing genes or proteins
- Supply context critical for comprehension prominently and in proximity
- Define abbreviations and acronyms the first time they are used
- Refer to concepts primarily by name, not description
- Choose a term for each concept and use it consistently
- Prefer the complete and precise scientific term
- Avoid creating complex coordinated compound terms
- Recheck for word spacing errors
- Verify parentheses and brackets are correctly paired
- Recheck for misspellings using a different method
